# Methylome profiling of healthy and central precocious puberty girls

**DOI:** 10.1186/s13148-018-0581-1

**Published:** 2018-11-22

**Authors:** Danielle S. Bessa, Mariana Maschietto, Carlos Francisco Aylwin, Ana P. M. Canton, Vinicius N. Brito, Delanie B. Macedo, Marina Cunha-Silva, Heloísa M. C. Palhares, Elisabete A. M. R. de Resende, Maria de Fátima Borges, Berenice B. Mendonca, Irene Netchine, Ana C. V. Krepischi, Alejandro Lomniczi, Sergio R. Ojeda, Ana Claudia Latronico

**Affiliations:** 1 Division of Endocrinology & Metabolism, Development Endocrinology Unit, Laboratory of Hormones and Molecular Genetics/LIM42, Clinical Hospital, Sao Paulo Medical School, University of Sao Paulo, Sao Paulo, SP Brazil; 2 Brazilian Biosciences National Laboratory (LNBio), Brazilian Center for Research in Energy and Materials (CNPEM), Campinas, SP Brazil; 3 Division of Genetics, Oregon National Primate Research Center/OHSU, Beaverton, OR USA; 4 Sorbonne Université, INSERM, UMR_S 938 Centre de Recherche Saint Antoine, APHP, Hôpital Armand Trousseau, Explorations Fonctionnelles Endocriniennes, Paris, France; 5 Division of Endocrinology, Triangulo Mineiro Federal University, Uberaba, MG Brazil; 6 Department of Genetics and Evolutionary Biology, Institute of Biosciences, University of Sao Paulo, Sao Paulo, SP Brazil; 7 Division of Neuroscience, Oregon National Primate Research Center/OHSU, Beaverton, OR USA; 8 Hospital das Clínicas, Faculdade de Medicina da Universidade de São Paulo, Departamento de Clínica Médica, Disciplina de Endocrinologia e Metabologia, Av. Dr. Enéas de Carvalho Aguiar, 255, 7° andar, sala 7037, São Paulo, CEP: 05403-900 Brazil

**Keywords:** Human puberty, Central precocious puberty, DNA methylation, Epigenetics, Genomic imprinting, Zinc finger genes

## Abstract

**Background:**

Recent studies demonstrated that changes in DNA methylation (DNAm) and inactivation of two imprinted genes (*MKRN3* and *DLK1*) alter the onset of female puberty. We aimed to investigate the association of DNAm profiling with the timing of human puberty analyzing the genome-wide DNAm patterns of peripheral blood leukocytes from ten female patients with central precocious puberty (CPP) and 33 healthy girls (15 pre- and 18 post-pubertal). For this purpose, we performed comparisons between the groups: pre- versus post-pubertal, CPP versus pre-pubertal, and CPP versus post-pubertal.

**Results:**

Analyzing the methylome changes associated with normal puberty, we identified 120 differentially methylated regions (DMRs) when comparing pre- and post-pubertal healthy girls. Most of these DMRs were hypermethylated in the pubertal group (99%) and located on the X chromosome (74%). Only one genomic region, containing the promoter of *ZFP57*, was hypomethylated in the pubertal group. *ZFP57* is a transcriptional repressor required for both methylation and imprinting of multiple genomic loci. *ZFP57* expression in the hypothalamus of female rhesus monkeys increased during peripubertal development, suggesting enhanced repression of downstream ZFP57 target genes. Fourteen other *zinc finger* (*ZNF*) genes were related to the hypermethylated DMRs at normal puberty. Analyzing the methylome changes associated with CPP, we demonstrated that the patients with CPP exhibited more hypermethylated CpG sites compared to both pre-pubertal (81%) and pubertal (89%) controls. Forty-eight *ZNF* genes were identified as having hypermethylated CpG sites in CPP.

**Conclusion:**

Methylome profiling of girls at normal and precocious puberty revealed a widespread pattern of DNA hypermethylation, indicating that the pubertal process in humans is associated with specific changes in epigenetically driven regulatory control. Moreover, changes in methylation of several *ZNF* genes appear to be a distinct epigenetic modification underlying the initiation of human puberty.

**Electronic supplementary material:**

The online version of this article (10.1186/s13148-018-0581-1) contains supplementary material, which is available to authorized users.

## Background

The onset of puberty is heralded by an increased pulsatile secretion of gonadotropin-releasing hormone (GnRH), which upon reaching the anterior pituitary activates the pituitary–gonadal axis. Epidemiological studies have provided evidence supporting a genetic influence on pubertal timing [1–3]. However, the age at normal puberty varies greatly among girls (8–13 years) and the genetic basis for such a variability remains largely unknown [4]. A potential underlying mechanism is the modulation of gene activity by epigenetic factors, which may be important for the broad regulation of pubertal timing [5]. In fact, it appears that up to 20% of the variance of puberty initiation involves environmental factors, such as nutrition, stress, exposure to endocrine-disrupting chemicals, and intrauterine conditions [5, 6].

Epigenetics refers to the alterations in gene expression that are not caused by changes in DNA sequence itself [7]. DNA methylation (DNAm) is one of the best studied epigenetic mechanisms involved in modulating gene activity [8, 9]. It consists of the covalent addition of a methyl (-CH3) group to the fifth position of the pyrimide base of DNA, cytosine, and occurs mostly in cytosine–phosphate–guanine (CpG) dinucleotides [8].

Epigenetics has been implicated as a regulatory system underlying GnRH secretion [10, 11]. The study of DNAm in the medial basal hypothalamus of male rhesus monkeys revealed a decrease in methylation status of the GnRH gene’s 5′ CpG island that paralleled an increase in GnRH mRNA levels across puberty [12]. Indeed, increased DNAm of gene promoters is commonly associated with gene silencing [13, 14]. Recently, silencers of the Polycomb group were identified as major drivers of an epigenetic mechanism of transcriptional repression that is lifted at the beginning of female puberty in rats, allowing the pubertal process to proceed unimpeded [10]. Importantly, manipulations of DNAm in animal models were shown to alter the onset of puberty. Thus, inhibiting DNAm resulted in pubertal failure, whereas inducing DNA hypermethylation led to earlier onset of puberty [10, 15]. DNAm also plays an essential role in genomic imprinting, an epigenetic phenomenon recently implicated in the regulation of puberty. Initial evidence for this concept came from studies showing that common variants located at the loci of three imprinted genes (*MKRN3*, *DLK1*, and *KCNK9*) were associated with the age at menarche in a large European women cohort [16]. More direct evidence was provided by the demonstration that central precocious puberty (CPP) due to loss-of-function mutations in the paternally expressed imprinted genes *MKRN3* (*makorin ring finger 3*) and *DLK1* (*delta-like 1 homolog*) is an imprinting disorder [17–19].

In the present study, we used peripheral blood leukocytes to investigate the relationship that may exist between DNAm patterns and pubertal timing in healthy and CPP-affected girls. We compared pre- with post-pubertal control subjects to interrogate changes in the methylome profile that occurs during physiological pubertal development. We also compared CPP patients with healthy girls to analyze the DNAm changes in CPP.

## Results

### Description of the analyzed human groups

We studied ten female patients with familial CPP (index cases) who were referred for clinical and/or genetic evaluation to the Endocrinology Unit at Clinical Hospital, Sao Paulo, Brazil. CPP in girls was diagnosed based on the presence of breast Tanner stage 2 (B2) before the age of 8 years, pubertal basal and/or GnRH-stimulated LH levels, and bone age advanced more than 1 year (Greulich and Pyle atlas). Clinical and hormonal features of the patients with CPP are described in Table 1. The mean age at pubertal onset of these girls was 6.4 years (ranging from 3 to 8 years). At the time of the first evaluation (mean age of 7.7 years), Tanner B3 was observed in 50% of the girls and Tanner B4 in the remaining 50%. The mean Δ [bone age − chronological age] was 2.2 ± 1.1 years. None of the patients were obese (mean body mass index (BMI) *Z*-score = 0.7). Mean basal LH levels were 1.4 ± 1.4 IU/L, and mean LH levels after GnRH stimulation were 18.9 ± 14 IU/L. Mean basal FSH levels were 3.4 ± 1.7 IU/L. Median estradiol (E2) levels were 13 pg/mL. All CPP patients had normal brain magnetic resonance imaging.

**Table 1:** Clinical and hormonal features of the patients with CPP

Family number	Initial clinical manifestation (age, y)	Time of diagnosis			BA (y)	LH (IU/L)			FSH (IU/L), Basal	E2 (pg/mL)
		Age (y)	Breast Tanner stage	BMI (*Z*)		Basal	After GnRH	After leuprolide depot 3.75 mg		
1	Telarche (5.8)	6.7	4	1.2	11	1.9	31.5	NA	6.2	53.3
2	Telarche (7.7)	8.2	3	0.09	11	< 0.6	NA	17.6	4.2	31
3	Telarche (5.5)	8.1	3	0.17	11	1	NA	8.8	1.9	17.4
4	Telarche (7.5)	10.2	4	1.29	11	4.2	NA	NA	NA	98.8
5	Telarche (7.3)	8	3	1.28	11	1.5	NA	45.8	2.7	< 13
6	Telarche (6.6)	7.3	4	1.41	9.5	0.1	16.9	16	NA	< 13
7	Telarche (5.5)	7.1	3	0.11	7.8	< 0.6	NA	6.5	1.5	< 13
8	Telarche (3)	6.5	4	0.64	8.8	< 0.6	4.2	9.8	3.7	< 13
9	Telarche (8)	8	4	− 0.09	10	0.1	6.9	4.6	2	< 13
10	Telarche (6.9)	6.9	3	0.85	7.8	3.5	35	NA	5.2	< 10

*Abbreviations*: *BA* bone age, *BMI* body mass index, *E2* estradiol, *NA* not available, *y* years

**Fig. 1:**
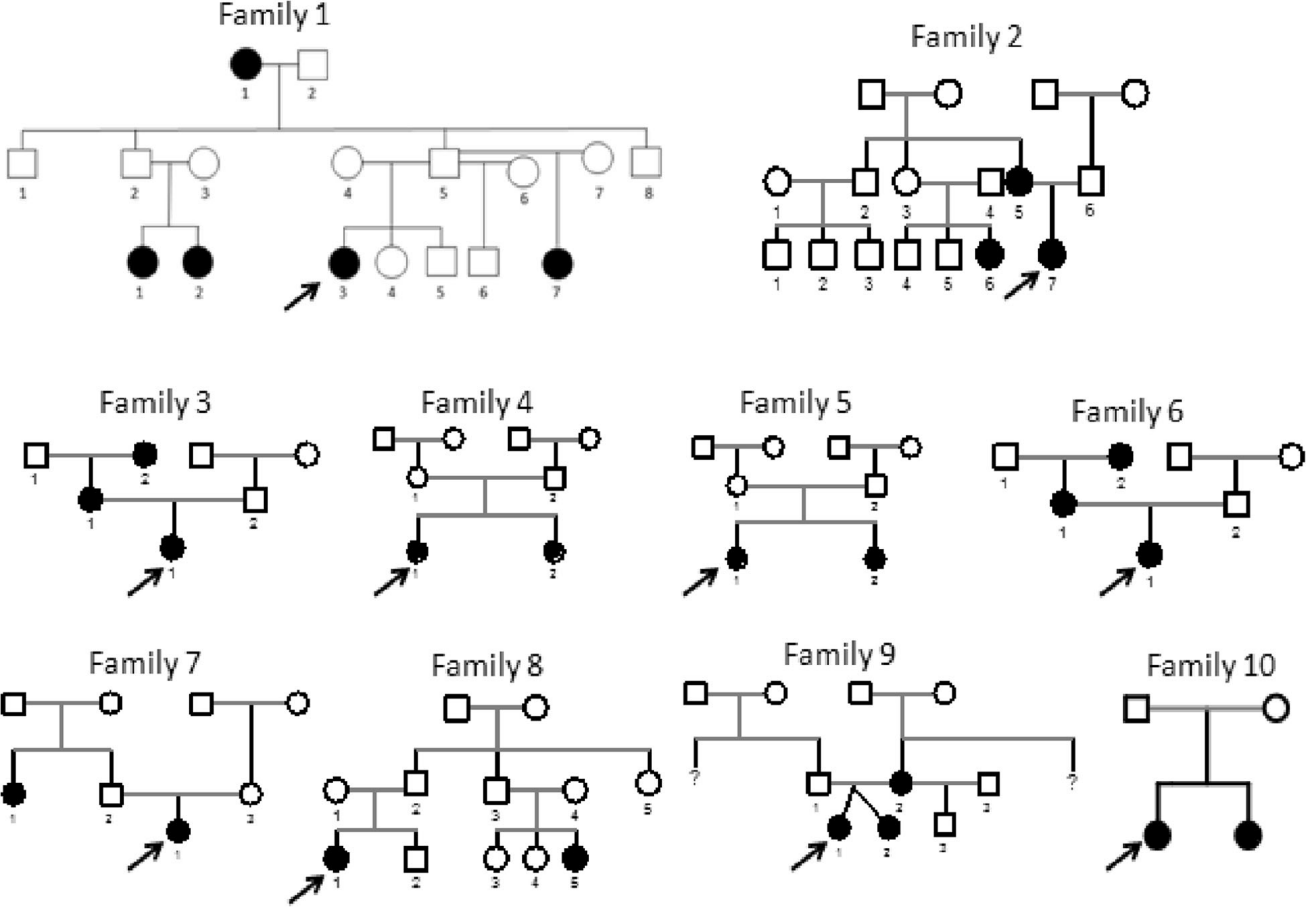
Pedigrees of the families with CPP. Squares indicate male members, circles female members, black symbols clinically affected members, and arrows the probands

Familial CPP was defined by the presence of more than one affected member in a family [2]. The pedigrees of the ten families are illustrated in Fig. 1. Only female members were affected in all families. Regarding the mode of inheritance, CPP was maternally inherited in four families (pedigrees 2, 3, 6, 9), paternally inherited in four families (pedigrees 1, 7, 8, 10), and undetermined in two families (pedigrees 4 and 5). A MKRN3 inactivating mutation (p.R328H) was detected in family 10 by Sanger sequencing. Whole-genome sequencing revealed a complex defect in *DLK1* (∼ 14 kb deletion and 269 bp duplication) in family 1 [18]. The remaining families (pedigrees 2 to 9) were previously studied by whole-exome sequencing without identifying a genetic mutation associated with CPP phenotype [17].

The control group was composed of 33 healthy Brazilian girls. Pubertal stage was characterized by physical signs (Tanner criteria) and hormonal evaluation (Tables 2 and 3). Fifteen of these girls were at pre-pubertal stage with mean chronological age of 6.7 years, ranging from 2.6 to 9 years. All of them exhibited Tanner B1 upon physical medical evaluation and pre-pubertal basal LH levels (< 0.1 IU/L). The mean BMI *Z*-score was 0.2 ± 0.6. The remaining 18 girls were at pubertal stage, with mean chronological age of 13.1 years, ranging from 9.5 to 16.3 years. The majority of them (61%) had Tanner B4. In the pubertal group, mean basal LH levels were 5.2 ± 2.4 IU/L, mean basal FSH levels were 5.4 ± 1.8 IU/L, and mean E2 levels were 67.6 ± 42.7 pg/mL. The mean BMI *Z*-score was 0.1 ± 0.7.

**Table 2:** Clinical and hormonal features of the pre-pubertal control group

Pre-pubertal control number	Chronological age (years)	BMI (*Z*)	Breast Tanner stage	Pubic hair Tanner stage	Basal LH (IU/L)	Basal FSH (IU/L)	E2 (pg/mL)
1	6.1	0.55	1	1	< 0.1	3	< 15
2	8.1	− 0.16	1	1	< 0.1	1.4	< 15
3	7.2	− 0.73	1	1	< 0.1	0.7	< 15
4	6.6	1.12	1	1	< 0.1	3.1	< 15
5	6.2	0.61	1	1	< 0.1	1.4	< 15
6	5.2	0.63	1	1	< 0.1	1.5	< 15
7	5.7	0.99	1	1	< 0.1	2.4	< 15
8	7.2	0.44	1	1	< 0.1	1.6	< 15
9	9	− 0.02	1	1	< 0.1	2.9	< 15
10	5.4	− 0.86	1	1	< 0.1	8.9	< 15
11	8.6	0.07	1	1	< 0.1	1.8	< 15
12	7.2	− 0.47	1	1	< 0.1	2.2	< 15
13	7.9	0.90	1	1	< 0.1	2.4	< 15
14	7.5	0.32	1	1	< 0.1	3.3	15.7
15	2.6	− 0.6	1	1	< 0.1	3.5	< 15

*Abbreviations*: *BMI* body mass index, *E2* estradiol

**Table 3:** Clinical and hormonal features of the pubertal control group

Pubertal control number	Chronological age (years)	BMI (*Z*)	Breast Tanner stage	Pubic hair Tanner stage	Basal LH (IU/L)	Basal FSH (IU/L)	E2 (pg/mL)
1	14.4	− 0.55	4	5	12	9.1	58
2	12.5	0.06	4	4	1.3	2.5	37.9
3	11.2	− 0.5	3	3	5.7	6.9	68.9
4	12	0.44	4	4	4.3	4.7	35.7
5	16.3	0.56	5	5	4.9	6.5	39.1
6	12.7	0.03	4	4	8.8	6.7	135.4
7	16	1.06	5	5	3.8	2.5	< 15
8	15.7	1.35	5	5	7.2	4.6	56.6
9	11.3	0.99	4	5	5.8	3.1	30.9
10	11.9	− 0.65	4	4	3.3	6.2	57.1
11	11.3	− 0.22	3	2	5.1	5.4	32.2
12	15.7	0.71	5	4	6.7	6.8	76
13	12.2	0.04	4	4	4.2	6.5	36.9
14	9.5	− 0.03	3	2	3.3	6.6	62.6
15	14.3	− 1.7	4	5	3.5	6.5	59.9
16	12.2	0.39	4	5	2.8	3	119.2
17	13.8	0.3	4	5	5.9	5.7	122.5
18	13.1	0.01	4	5	5.2	4.7	173.6

*Abbreviations*: *BMI* body mass index, *E2* estradiol

### Changes in DNA methylation associated with normal puberty

Comparison between pre-pubertal and pubertal healthy girls revealed the presence of 120 differentially methylated regions (DMRs) (false discovery rate (FDR) < 0.05 and methylation differences > 5%), with all but one (99%) being hypermethylated in the pubertal group (Table 4). Most of the DMRs (89 DMRs, 74%) were located on the X chromosome, none of them mapped to the pseudoautosomal regions (PAR) of this chromosome. The 120 DMRs harbored the promoter regions of 127 genes, whose functions were enriched for various biological processes, such as intracellular receptor signaling pathway, messenger RNA (mRNA) transcription, histone modification, and genetic imprinting (Additional file 1).

**Table 4:** Genomic regions with methylation differences > 5% between pre- and post-pubertal control groups

DMR identification	Chromosome	DMR start	DMR end	Number of CpGs	Mean beta difference (pre-pubertal minus post-pubertal values)	Promoters of genes included in the genomic region
DMR_1372	10	42,862,876	42,863,594	9	− 0.105162452	*RP11-313 J2.1*
DMR_8814	4	81,117,647	81,119,473	11	− 0.089040315	*PRDM8*
DMR_4264	16	1,583,810	1,584,516	8	− 0.084174074	*IFT140*, *TMEM204*
DMR_11738	X	48,814,205	48,815,856	16	− 0.08181042	*OTUD5*, *RNU6-722P*
DMR_9935	6	31,148,332	31,148,748	14	− 0.078236383	*POU5F1*
DMR_12001	X	153,671,800	153,672,898	8	− 0.076624259	*FAM50A*, *GDI1*
DMR_11933	X	132,090,860	132,092,772	7	− 0.075099028	*HS6ST2*
DMR_11979	X	152,066,221	152,066,735	7	− 0.073224659	NA
DMR_11993	X	153,598,742	153,600,494	7	− 0.072362363	*FLNA*
DMR_11972	X	149,861,526	149,862,363	7	− 0.070984952	*MTMR1*
DMR_9959	6	31,650,735	31,651,362	18	− 0.070185244	*LY6G5C*
DMR_10023	6	33,047,185	33,049,505	22	− 0.069255178	*HLA-DPB1*, *HLA-DPA1*, *RPL32P1*
DMR_11751	X	49,643,431	49,644,706	7	− 0.067077238	*USP27X-AS1*, *USP27X*
DMR_11804	X	71,130,538	71,131,891	7	− 0.066738144	*NHSL2*
DMR_11950	X	135,578,793	135,580,181	18	− 0.063932352	*HTATSF1*
DMR_7255	20	5,485,144	5,485,511	8	− 0.062564433	*LINC00654*
DMR_3109	12	130,823,570	130,824,831	8	− 0.062155177	*PIWIL1*
DMR_11802	X	70,712,403	70,713,213	8	− 0.0621177	*INGX*, *Y_RNA.73*, *TAF1*
DMR_11934	X	133,118,088	133,119,961	11	− 0.061748623	*GPC3*
DMR_12006	X	153,774,721	153,776,358	18	− 0.061612111	*IKBKG*, *G6PD*
DMR_5381	17	78,865,087	78,865,755	7	− 0.061329072	NA
DMR_11732	X	48,554,185	48,555,977	7	− 0.061177896	*SUV39H1*
DMR_11995	X	153,605,635	153,607,981	9	− 0.061083719	*EMD*
DMR_11921	X	129,114,238	129,118,953	19	− 0.060957678	*BCORL1*
DMR_11500	8	144,789,164	144,790,772	9	− 0.060451112	*ZNF707*, *CCDC166*
DMR_9283	5	77,145,356	77,147,141	8	− 0.060227285	NA
DMR_11790	X	68,723,670	68,725,815	13	− 0.06007949	*FAM155B*, *AL158069.1*
DMR_11669	X	21,391,817	21,393,898	12	− 0.059401453	*CNKSR2*
DMR_11823	X	79,590,789	79,591,032	8	− 0.059246208	*FAM46D*
DMR_11821	X	77,358,961	77,360,510	14	− 0.059121003	*PGK1*
DMR_11666	X	19,533,022	19,534,066	10	− 0.058953271	*MAP3K15*
DMR_11670	X	21,674,815	21,677,134	14	− 0.058921343	*KLHL34*
DMR_11678	X	24,482,846	24,484,361	10	− 0.058918654	*PDK3*
DMR_11711	X	46,432,770	46,434,442	9	− 0.058894854	*CHST7*
DMR_9843	6	28,601,271	28,601,519	11	− 0.05882722	*RP11-373 N24.2*
DMR_11966	X	147,581,562	147,583,550	15	− 0.058723364	*AFF2*, *AC002368.4*
DMR_11659	X	17,392,798	17,393,584	7	− 0.058571815	*NHS*
DMR_11900	X	119,005,413	119,006,837	14	− 0.058548975	*NDUFA1*, *RNF113A*
DMR_11742	X	48,931,388	48,932,122	11	− 0.058333756	*PRAF2*, *WDR45*
DMR_12004	X	153,718,691	153,719,406	10	− 0.058126507	*SLC10A3*
DMR_11999	X	153,656,860	153,657,411	7	− 0.058031895	*BX936347.1*, *ATP6AP1*
DMR_11748	X	49,056,505	49,057,013	9	− 0.058008258	*SYP*, *SYP-AS1*
DMR_2557	12	9,217,079	9,217,907	11	− 0.057945682	*A2M-AS1*, *LINC00612*
DMR_11970	X	149,529,976	149,534,258	17	− 0.057730577	*MAMLD1*
DMR_5006	17	37,123,638	37,124,558	10	− 0.057306128	*FBXO47*
DMR_6288	19	54,040,774	54,041,856	10	− 0.057304041	*ZNF331*
DMR_11944	X	134,232,157	134,233,109	7	− 0.057092491	*LINC00087*
DMR_11957	X	138,285,393	138,287,900	17	− 0.056982737	*FGF13*
DMR_11888	X	118,107,540	118,110,766	16	− 0.056978196	*LONRF3*
DMR_11764	X	54,383,906	54,385,662	7	− 0.056970851	*WNK3*
DMR_12003	X	153,713,930	153,715,426	10	− 0.05672695	*UBL4A*
DMR_11994	X	153,602,509	153,603,503	11	− 0.056715308	*FLNA*
DMR_11991	X	153,284,899	153,285,934	7	− 0.056647889	*IRAK1*, *MIR718*
DMR_11693	X	38,420,326	38,421,503	11	− 0.056490464	*TSPAN7*
DMR_11668	X	20,283,423	20,286,779	12	− 0.056376843	*RPS6KA3*
DMR_11672	X	21,958,416	21,959,700	10	− 0.056271754	*SMS*, *LL0XNC01-39B3.1*
DMR_1702	10	123,355,268	123,358,317	17	− 0.056225675	*FGFR2*
DMR_11974	X	150,150,670	150,153,136	17	− 0.056110647	*HMGB3*
DMR_11919	X	128,977,299	128,978,347	14	− 0.055916881	*ZDHHC9*
DMR_11770	X	55,187,242	55,187,903	10	− 0.055775168	*FAM104B*
DMR_7483	20	57,425,157	57,428,473	65	− 0.05557006	*GNAS*, *GNAS-AS1*
DMR_9132	5	23,507,030	23,507,656	7	− 0.055437765	*PRDM9*
DMR_11978	X	151,999,239	152,000,347	12	− 0.055139575	*NSDHL*, *CETN2*
DMR_12000	X	153,664,475	153,665,799	9	− 0.055090146	*GDI1*
DMR_11729	X	48,432,279	48,433,876	12	− 0.054825597	*RBM3*, *AC115618.1*
DMR_10691	7	50,849,168	50,851,503	9	− 0.054780529	*GRB10*
DMR_11873	X	107,334,429	107,335,227	11	− 0.054776826	*PSMD10*, *ATG4A*
DMR_11984	X	152,906,667	152,910,369	14	− 0.054741445	*DUSP9*
DMR_11642	X	11,774,782	11,777,794	18	− 0.054708295	*MSL3*
DMR_1209	1	240,656,217	240,657,329	8	− 0.054513802	NA
DMR_11894	X	118,707,913	118,709,261	14	− 0.054314233	*UBE2A*
DMR_11684	X	30,326,328	30,327,819	14	− 0.053883806	*NR0B1*
DMR_4794	17	6,898,738	6,899,888	15	− 0.053689222	*ALOX12*, *RP11-589P10.5*
DMR_11998	X	153,639,287	153,640,967	12	− 0.053584581	*DNASE1L1*, *TAZ*
DMR_11725	X	47,862,977	47,863,707	8	− 0.05357646	*SPACA5*, *ZNF182*
DMR_11648	X	15,353,254	15,354,150	12	− 0.053561291	*PIGA*
DMR_11911	X	122,993,419	122,994,594	14	− 0.053524318	*XIAP*
DMR_11743	X	48,957,691	48,958,509	12	− 0.053409715	*WDR45*
DMR_11741	X	48,900,948	48,901,982	9	− 0.053374186	*TFE3*
DMR_11915	X	128,656,725	128,657,893	11	− 0.053357781	*SMARCA1*
DMR_7894	22	51,016,501	51,017,151	12	− 0.053231588	*CPT1B*, *CHKB-CPT1B*, *CHKB*
DMR_11942	X	134,165,954	134,166,347	7	− 0.053015868	*FAM127A*
DMR_11720	X	47,478,591	47,479,662	11	− 0.052859687	*SYN1*
DMR_11777	X	62,974,433	62,975,657	10	− 0.052826713	*ARHGEF9*
DMR_11903	X	119,148,809	119,150,375	8	− 0.052776664	*GS1-421I3.2*
DMR_11918	X	128,913,578	128,913,980	7	− 0.052666423	*SASH3*
DMR_11923	X	129,243,062	129,246,153	12	− 0.05258674	*ELF4*
DMR_10704	7	56,515,510	56,516,504	10	− 0.052581782	*RP13-492C18.2*
DMR_11996	X	153,625,785	153,628,595	17	− 0.052516898	*RPL10*, *SNORA70*
DMR_12002	X	153,707,029	153,708,103	8	− 0.052282548	*LAGE3*
DMR_11892	X	118,601,891	118,603,378	9	− 0.052141155	*SLC25A5*, *SLC25A5-AS1*
DMR_11890	X	118,369,571	118,370,873	12	− 0.05200566	*PGRMC1*
DMR_11977	X	151,806,225	151,807,197	11	− 0.051997479	*GABRQ*
DMR_11781	X	64,254,409	64,255,552	7	− 0.051933195	*ZC4H2*
DMR_9869	6	29,629,187	29,631,447	10	− 0.051487467	NA
DMR_11852	X	101,966,398	101,967,643	8	− 0.051470117	*GPRASP2*
DMR_11718	X	47,341,740	47,343,198	13	− 0.051358132	*ZNF41*, *CXorf24*
DMR_11677	X	23,925,454	23,927,284	12	− 0.051200853	*CXorf58*, *APOO*
DMR_9823	6	28,058,715	28,059,208	9	− 0.051119312	*ZSCAN12P1*
DMR_11703	X	41,781,891	41,783,785	10	− 0.05111124	*CASK*
DMR_11675	X	23,760,460	23,762,372	13	− 0.051058352	*ACOT9*
DMR_11714	X	47,003,362	47,004,911	16	− 0.051047155	*NDUFB11*, *RBM10*
DMR_11834	X	99,661,860	99,667,528	23	− 0.051019999	*PCDH19*
DMR_9036	4	174,421,114	174,422,908	9	− 0.050942397	NA
DMR_11955	X	136,647,133	136,649,808	10	− 0.050903123	*ZIC3*, *RP1-137H15.2*
DMR_11909	X	119,763,444	119,764,469	11	− 0.050899975	*C1GALT1C1*
DMR_10849	7	100,463,206	100,465,221	11	− 0.050845841	*TRIP6*
DMR_4309	16	3,493,133	3,494,155	12	− 0.050709712	*ZNF597*, *NAA60*
DMR_11695	X	38,662,279	38,665,168	16	− 0.050532132	*MID1IP1*, *MID1IP1-AS1*
DMR_11785	X	67,653,156	67,653,925	10	− 0.050494689	*OPHN1*
DMR_12009	X	154,032,629	154,034,184	11	− 0.050494431	*MPP1*
DMR_11922	X	129,193,893	129,194,982	7	− 0.050454357	NA
DMR_5488	18	14,747,888	14,748,439	10	− 0.050398645	*ANKRD30B*
DMR_11851	X	101,905,837	101,907,254	8	− 0.050370988	*GPRASP1*
DMR_12011	X	154,299,274	154,300,204	8	− 0.050341015	*BRCC3*, *MTCP1*, *CMC4*
DMR_4813	17	7,311,030	7,312,081	9	− 0.05032058	*NLGN2*
DMR_11673	X	23,350,093	23,353,620	13	− 0.050150411	*PTCHD1*
DMR_11763	X	54,209,148	54,209,968	8	− 0.050134574	*FAM120C*
DMR_11902	X	119,133,852	119,135,074	7	− 0.050060465	NA
DMR_9870	6	29,648,161	29,649,084	22	0.08795856	*ZFP57*

*Abbreviations*: *DMR* differentially methylated region, *NA* not available

The single hypomethylated genomic region identified in pubertal girls encompassed the promoter region of *ZFP57* (*zinc finger protein 57*), a KRAB domain-containing transcriptional repressor involved in both imprinting and methylation of multiple genomic loci [20, 21]. The DMR with the largest methylation difference (10.5%) contained the promoter region of *RP11-313 J2.1*, the zinc finger protein 91 pseudogene. In addition, the promoter regions of 13 other *zinc finger* (*ZNF*) genes (*ZNF597*, *ZSCAN12P1*, *ZNF707*, *ZNF331*, *ZC4H2*, *ZNF182*, *ZNF41*, *ZIC3*, *RNF113A*, *ZDHHC9*, *PRDM8*, *PRDM9*, *RBM10*) were related to these 120 DMRs.

We applied the Gene Set Enrichment Analysis (GSEA) to search for enriched transcription factors that could be targeting the identified DMRs. We detected enrichment for 20 transcription factors, and the ten most relevant are listed in Additional file 2. Importantly, one of them is the estrogen receptor (ER). Seven differentially methylated genes (*SMARCA1*, *POU5F1*, *HTATSF1*, *ELF4*, *HMGB3*, *KLHL34*, *FGFR2*) had ER binding sites in the region spanning up to 4 kb around their transcription start site (TSS).

### Changes in DNA methylation associated with CPP

A unique DMR was detected between CPP cases and pre-pubertal controls (FDR < 0.05), and it was slightly more methylated in the CPP group (mean beta difference of 0.003391969). This genomic region (chr6: 33385679-33385786) harbored the promoter region of *CUTA* (homolog of *Escherichia coli* CutA), a gene ubiquitously expressed, including brain. Comparison between CPP cases and pubertal girls revealed the absence of DMRs (FDR < 0.05). Because of this, we explored the methylation levels at isolated CpG sites.

**Fig. 2:**
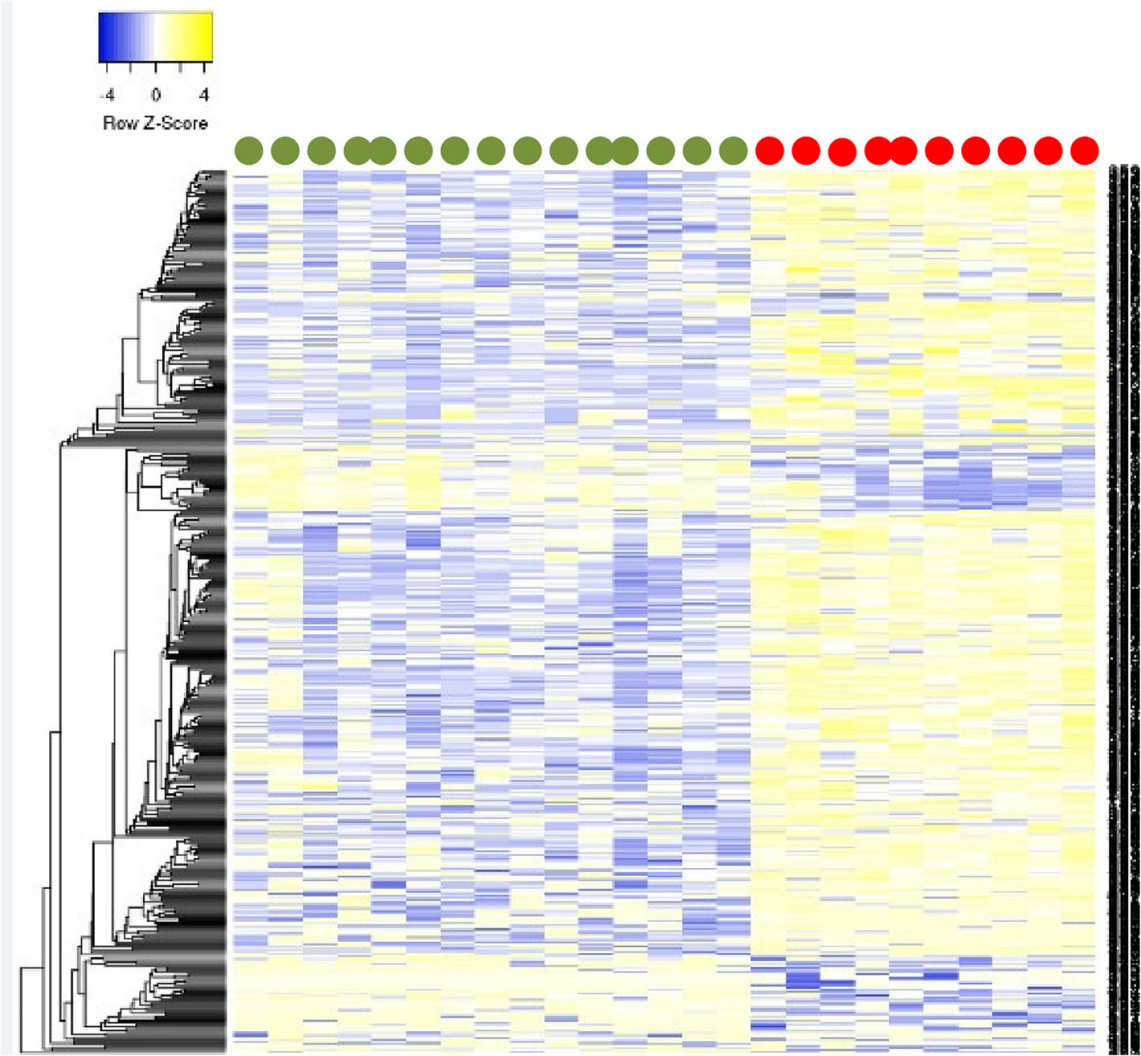
Heatmap based on the methylation levels of the 417 DMSs identified between CPP cases and pre-pubertal controls. Cases are indicated in red and controls in green. Each column represents an individual, and each row represents a CpG site. Methylation levels are displayed in *Z*-score at the up left corner of the heatmap. Hierarchical clustering was applied to the CpG sites (rows) using Euclidian distance with average linkage

**Fig. 3:**
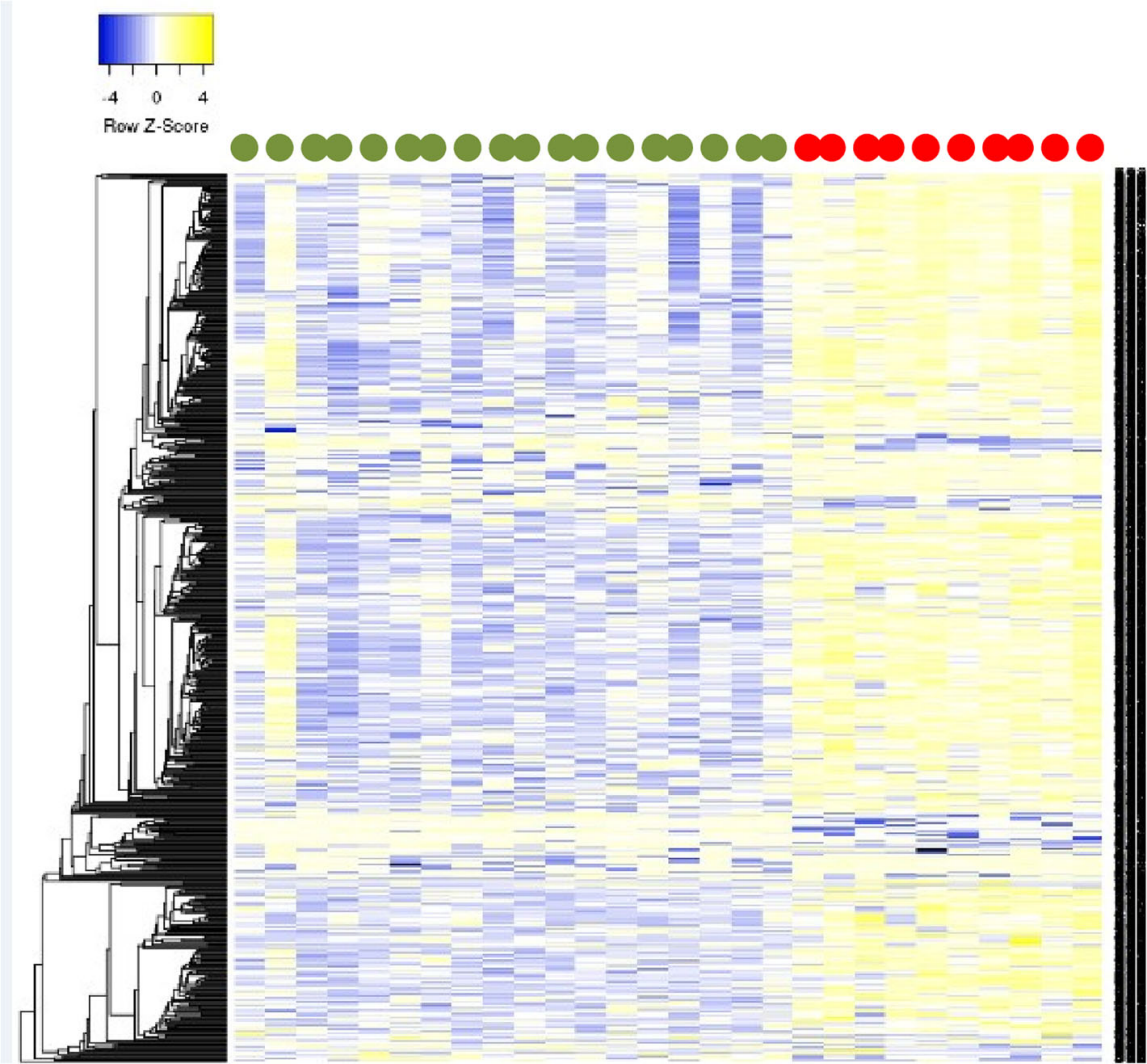
Heatmap based on the methylation levels of the 605 DMSs identified between CPP cases and post-pubertal controls. Cases are indicated in red and controls in green. Each column represents an individual, and each row represents a CpG site. Methylation levels are displayed in *Z*-score at the up left corner of the heatmap. Hierarchical clustering was applied to the CpG sites (rows) using Euclidian distance with average linkage

Comparison between CPP cases and pre-pubertal controls revealed 417 differentially methylated CpG sites (DMSs) (FDR < 0.05 and methylation differences > 10%), with the majority of them (338 DMSs, 81%) being hypermethylated in CPP patients (Fig. 2, Additional files 3 and 4). In silico functional analyses of the 199 known genes related to these 338 DMSs demonstrated enrichment for signaling pathways involved in cell communication (70 genes), regulation of response to stimuli (40 genes), and metabolism (10 genes). When comparing CPP cases with pubertal controls, we identified 605 DMSs (FDR < 0.05 and methylation differences > 10%), with the majority of them (539 DMSs, 89%) being hypermethylated in the CPP group (Fig. 3, Additional files 5 and 6). The functional characterization of 308 known genes related to these 539 DMSs revealed enrichment for metabolic pathways (13 genes), transport vesicles (10 genes), association with endocrine system diseases (10 genes), and carcinomas (13 genes). Forty-eight genes harboring hypermethylated CpG sites in CPP were *ZNFs* (Additional files 4 and 6).

### Normal methylation of the *MKRN3* and *DLK1* genes

Methylation status of the *MKRN3* and *DLK1* genes and their regulatory regions using two distinct methods revealed no differences between CPP patients and controls. The methylation analyses of *MKRN3*:TSS-DMR and *MEG3/DLK1*:IG-DMR (IG = intergenic) are showed in Additional file 7.

### Hypothalamic expression of *ZFP57* increases at puberty in nonhuman primates

**Fig. 4:**
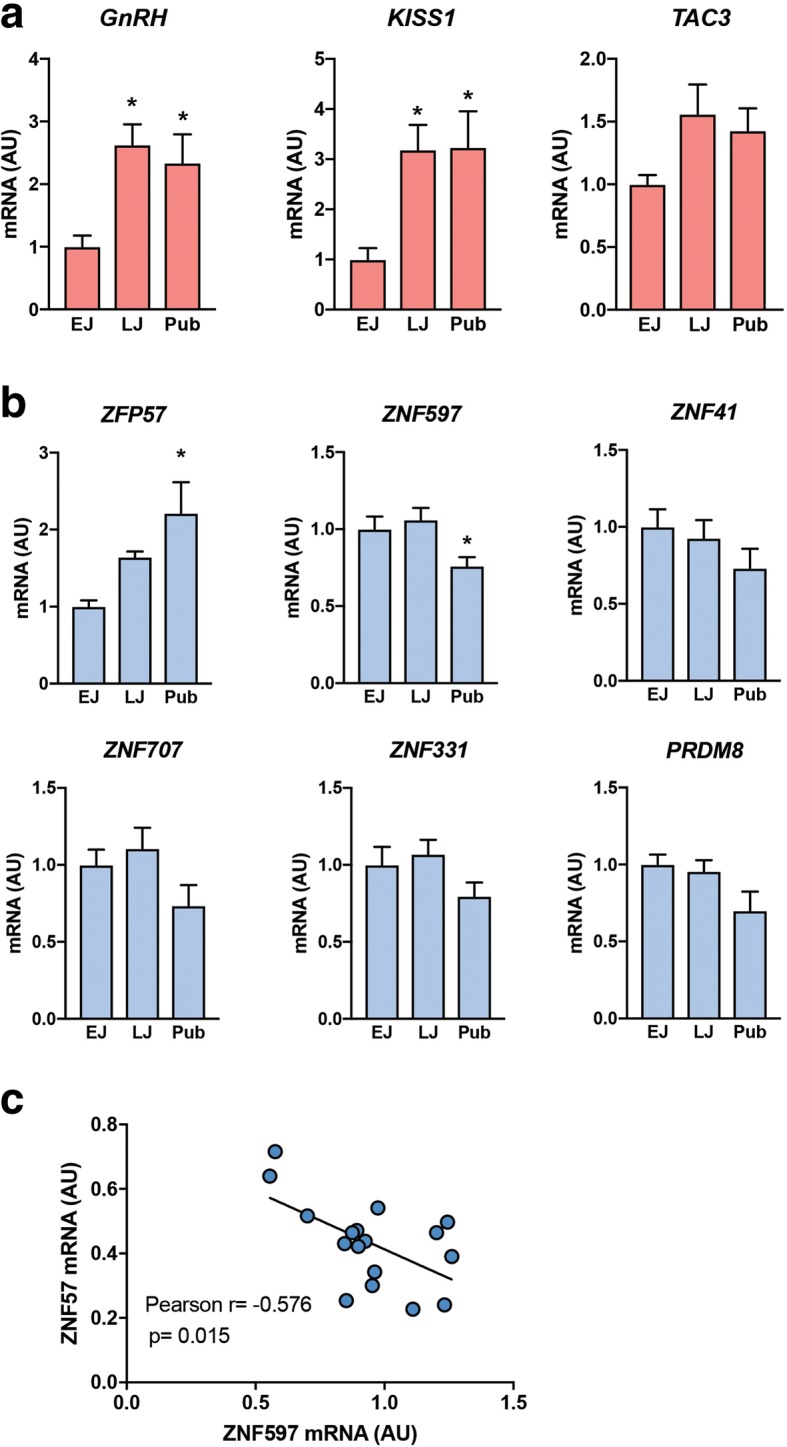
Changes in expression of **a** *GnRH*, *KISS1*, and *TAC3* and **b** selected *ZNFs* in the female monkey hypothalamus during pubertal development. One of the *ZNFs* examined (*ZFP57*) was hypomethylated at puberty in peripheral blood cells. The other five were hypermethylated. **c** Correlation analysis shows that a loss in *ZNF597* expression observed at the time of monkey puberty is negatively correlated to the increase in *ZFP57* mRNA levels detected at this time. The mRNA levels obtained were expressed as fold-change with regard to the values observed in the EJ group. Bars represent mean ± s.e.m. (*n* = 4–7/group) (*p < 0.05; vs EJ group; one-way ANOVA-SNK test). EJ early juvenile, LJ late juvenile, PUB peripubertal

To determine if hypomethylation of *ZFP57* in peripheral blood leukocytes is accompanied by increased *ZFP57* expression in the hypothalamus at the time of puberty, we measured *ZFP57* mRNA levels in the medial basal hypothalamus (MBH) of pre- and peripubertal female rhesus monkeys. We observed that *ZFP57* mRNA levels began to increase during late juvenile development and became significantly elevated at puberty, coinciding with the increase in *GnRH* and *KISS1* expression that occurs at this time (Fig. 4a, b). To determine if a decrease in expression of *ZNFs* that become hypermethylated at puberty also occurs at puberty in the monkey hypothalamus, we selected five of these genes for mRNA measurement. Interestingly, expression of all five genes showed a tendency to decrease at puberty (Fig. 4b), with the change in *ZNF597* being statistically significant. To further evaluate these results, we performed a correlation analysis of the changes in *ZFP57* and *ZNF597* expression that occurred with the advent of puberty and found the existence of a significant (*p* = 0.01) inverse correlation between the pubertal increase in *ZFP57* mRNA levels and the decrease in *ZNF597* expression (Fig. 4c). We also used the MBH of female rhesus monkeys to quantitate the hypothalamic expression of four *ZNFs* that become hypermethylated in CPP and found that their mRNA levels either increase at normal puberty (*ZNF251*) or showed no change (*RNF113A*, *ZDBF2*, and *ZDHHC9*) (Additional file 8a). This result is in keeping with the finding that *ZNFs* hypermethylated at CPP are not the same as those *ZNFs* that become hypermethylated at normal puberty.

In addition to defining the methylation status of *MKRN3* and *DLK1* in peripheral blood leukocytes of CPP patients and control subjects undergoing normal puberty, we examined the changes in *MKRN3* and *DLK1* mRNA levels that occur in the MBH of female monkeys at the time of puberty. No changes in expression for either gene were detected between the early juvenile and the pubertal phases of monkey puberty (Additional file 8b).

**Fig. 5:**
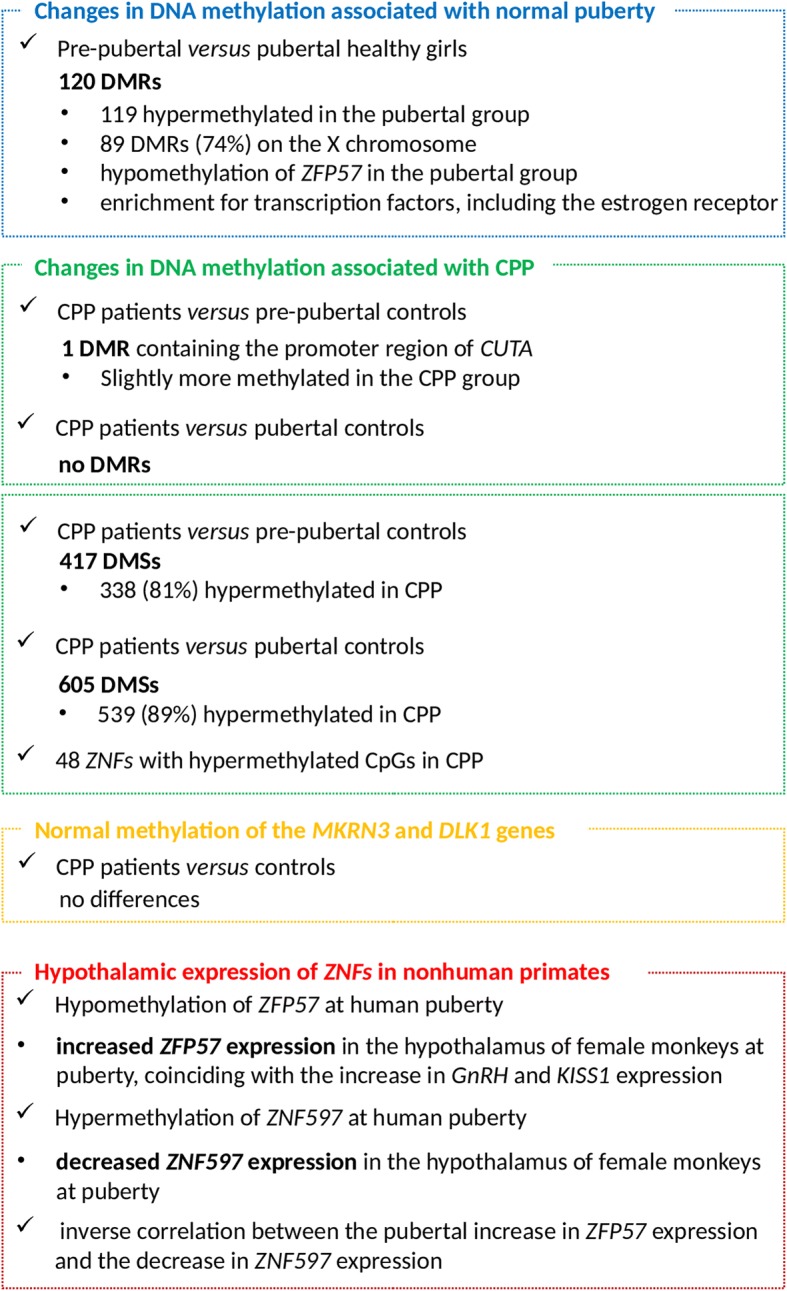
Flow chart summarizing the main results of the present study

Figure 5 summarizes the main results of the present study.

## Discussion

In the last few years, it has become increasingly clear that epigenetic mechanisms contribute significantly to the regulation of pubertal timing. From a clinical perspective, it would be desirable to have minimally invasive methods for the identification and monitoring of at least some of these mechanisms. Here, we report the use of white blood cells to assess patterns of DNAm that occur in association with puberty in girls. Our findings reveal the existence of a broad pattern of DNA hypermethylation taking place in these cells at the time of both normal and central precocious puberty. These findings are consistent with an earlier report showing an increase in DNAm levels of peripheral blood cells during the puberty transition in girls [22].

Most of the changes we observed consisted of hypermethylation of either DMRs or DMSs, with *ZNFs* standing out as a population of transcriptional repressors affected by these alterations. The sole exception to this pattern was *ZFP57*, a transcriptional repressor required for methylation of downstream genes and imprinting of several other genes [20, 23]. Contrary to other *ZNFs*, *ZFP57* was hypomethylated at puberty, suggesting that its expression may increase—instead of decrease—in tissues relevant to the pubertal transition. Measurement of *ZFP57* expression in the hypothalamus of female rhesus monkeys undergoing puberty proved this assumption to be correct, as a significant increase in *ZFP57* mRNA levels was detected at the time of puberty.

The *ZFP57* gene, located at chromosome 6p22.1, encodes a protein of 516 amino acids that contains seven zinc finger motifs [21, 24]. The interaction of these motifs with specific DNA sequences in regulatory regions of target genes is required for ZNF proteins to control gene expression [24]. Importantly, in the absence of *ZFP57*, genomic imprinting is lost [20]. This is illustrated by the loss of differential DNA methylation at the imprinted regions *Dlk1-Dio3* and *Snrpn* in homozygous mutant embryos (maternal-zygotic) derived from *Zfp57* null female mice [20]. Notably, directly relevant to the present findings, *ZFP57* has been shown to be required for normal imprinting of genomic regions controlling the expression of *MKRN3* and *DLK1*, two genes encoding repressors of the pubertal process, with *DLK1* being one of the genes most strongly affected by the absence of *ZFP57* [20]. The increase of *ZFP57* expression observed in the hypothalamus of pubertal female monkeys suggests that an important function of *ZFP57* in the neuroendocrine brain might be to repress the activity of transcriptional repressors of puberty, such as the Polycomb complex or other *ZNFs* [10, 25]. Furthermore, the inverse correlation detected between the increase in *ZFP57* mRNA levels and the decrease in *ZNF597* expression that occurred in the hypothalamus at the time of monkey puberty suggests that ZNF597 might be one of the transcriptional inhibitors of primate puberty repressed by ZFP57. Further studies are required to test the validity of this idea.

It is now known that pubertal timing requires repression of inhibitory factors and that DNA hypermethylation of gene promoters is associated with gene silencing [11, 26, 27]. In humans, genome-wide association studies revealed associations between single-nucleotide polymorphisms located near *ZNF131*, *ZNF462*, and *ZNF483* and earlier age of menarche, suggesting that *ZNF* genes can impact human pubertal development [16, 25, 28]. Supporting this concept are the demonstrations that *MKRN3*, also known as *ZNF127*, inhibits the human pubertal process and that *MKRN3* loss-of-function mutations are the most frequent cause of familial CPP [29–31]. More recently, the *DLK1* gene was also proposed to play an inhibitory role in the regulation of puberty, since its deficiency was associated with a CPP phenotype in syndromic and nonsyndromic cases [18, 32]. Within this context, our results showing a broad pattern of DNA hypermethylation at puberty suggest that—if hypermethylation of *ZNFs* also occurs in neuroendocrine cells controlling reproductive development—*ZNF* expression would decrease, and downstream target genes would escape from *ZNF* inhibitory control at the time of puberty. A specific example of this epigenetic interaction was recently provided by the demonstration that expression of several *ZNFs* decreases at puberty in the hypothalamus of female nonhuman primates and that preventing this change delayed pubertal timing [25]. In the present study, we measured the mRNA levels of five of these hypermethylated genes (*ZNF597*, *ZNF41*, *ZNF707*, *ZNF331*, and *PRDM8*) in de hypothalamus of developing female monkeys and found that all of them showed a tendency to decrease expression at the end of pubertal development, with the changes in *ZNF597* being significant.

As indicated above, there were 14 *ZNFs* hypermethylated at normal puberty. Intriguingly, the largest methylation difference (10.5%) was related to the zinc finger protein 91 pseudogene. At present, we do not know if it is involved in the hypothalamic control of puberty, but such a role remains possible, especially considering that 2–20% of human pseudogenes are transcribed, with some being transcribed in a tissue-specific manner maintained over the years [33]. It is therefore plausible that pseudogenes have a functional role in specific cell populations, an idea supported by the finding that noncoding RNAs produced from pseudogenes can regulate gene expression [33].

The search for transcription factors targeting the 120 DMRs associated with the pubertal process revealed an enrichment for ER. We identified seven differentially methylated genes as ER target genes, suggesting the existence of a functional relationship between them. This relationship appears to be particularly relevant to the neuronal regulation of the pubertal process, as neuronal ERα is involved in the temporal coordination of GnRH secretion, and an inhibitory ER-mediated influence on kisspeptin neurons has been shown to keep puberty in check in female mice [34]. Recently, the methylome study of 30 girls identified changes in DNAm across puberty related to estrogen-responsive genes, suggesting that differential DNAm at puberty may in part result from exposure to pubertal levels of estradiol [35].

An intriguing finding of this study was the striking prevalence of X-linked DMRs related to puberty. All the 89 X-linked DMRs were mapped outside the PAR regions of the X chromosome. PAR are short regions of homology between the mammalian X and Y chromosomes which are located at the tips of the short arm (PAR1) and long arm (PAR2) of the chromosome, and they harbor genes that escape X-inactivation [36]. Most of X-linked genes are subject to X-inactivation in females to ensure dosage compensation [36]. However, 15–20% of X-chromosomal genes escape from inactivation, and 80% of them lie on the short arm [37, 38]. Moreover, these escaping genes can have different expression levels between tissues and between females [38, 39]. Of note, some of the X-linked DMRs related to puberty identified in our study affect genes that escape X-inactivation, as *MSL3*, *NR0B1*, *RBM3* e *HS6ST2*, and others that are heterogeneous in escape, as *FGF13* e o *SLC25A5* [38]. The potential contribution of genes that escape X-inactivation to the timing of puberty was previously noticed. A case in point are the reports of girls with trisomy X (47, XXX karyotype) manifesting precocious puberty [40, 41]. Early activation of the hypothalamic–pituitary–gonadal axis in these girls was attributed to the extra X chromosome and more specifically to the expression of genes that escape X-inactivation. Early puberty was also described in females with Xp.11.22-p11.23 duplication [42]. In these patients, the duplicated X is preferentially activated, probably contributing to their clinical phenotype. Our results now demonstrate that changes in methylation of genes that escape X-inactivation occur in puberty.

A Danish study identified the methylation of a region on chromosome 7, which contains the promoter of *TRIP6* (thyroid hormone receptor interactor 6), to be associated with human pubertal development [43]. Our results support and extend these earlier findings by demonstrating an overlap of ten DMRs with the reported data, with seven presenting methylation changes in the same direction, including the DMR containing the *TRIP6* promoter (Table 5).

**Table 5:** DMRs related to pubertal process in this study that were previously reported by Almstrup during transition to puberty

DMR	Chr	DMR start	DMR end	DMR width	Number of CpGs	Mean beta difference (pre-pubertal minus post-pubertal values)	Minimum FDR	Promoters of genes included in the DMR
DMR_10849*	7	100,463,206	100465221	2016	11	− 0.050845841	8.06E−11	*TRIP6*
DMR_10848*	7	100,449,647	100,450,634	988	8	− 0.011123245	8.92E−22	*SLC12A9*, *RP11-126 L15.4*
DMR_4835	17	7,834,607	7,835,519	913	13	− 0.004918456	2.27E−35	*TRAPPC1*, *KCNAB3*, *CNTROB*
DMR_5149	17	4,6687,312	46,692,859	5548	34	− 0.010869697	1.33E−51	*HOXB8*, *HOXB7*
DMR_6184	19	46,996,347	46,999,444	3098	15	− 0.017502407	1.01E−10	*AC011484.1*, *PNMAL2*
DMR_9947*	6	31,543,289	31,543,686	398	10	− 0.024464641	1.26944E−07	*TNF*
DMR_5887*	19	15,528,530	15,530,737	2208	18	− 0.007917542	1.19E−30	*AKAP8L*
DMR_1838*	11	2,925,594	2,927,247	1654	15	0.005243223	2.57836E−07	*SLC22A18*, *SLC22A18AS*
DMR_9898*	6	30,290,258	30,296,049	5792	57	− 0.014466232	3.04E−79	*TRIM39*, *HCG18*
DMR_2718*	12	52,626,427	52,627,576	1150	10	− 0.024084513	1.47E−20	*KRT7*

*Abbreviations*: *Chr* chromosome, *DMR* differentially methylated regions, *FDR* false discovery rate

*DMRs with methylation changes in the same direction

Our study unveils a genome-wide DNA hypermethylation in CPP, which is in accordance with animal studies [15]. To our knowledge, this is the first study describing changes in the methylome patterns of girls with CPP. Hypermethylated CpGs in 63 genes were identified in CPP patients, including 48 *ZNF* genes. We speculate that these genes can either contribute to CPP or represent epigenetic modifications resulting from functional changes affecting the complex genetic network underlying the CPP disease. Although we cannot distinguish between these two possibilities, we notice the absence of significant DMRs between pubertal healthy girls and CPP patients. This suggests that the main epigenetic modifications that modulate gene expression during puberty, either normal or precocious, are similar. However, this conclusion is tempered by the finding that differentially methylated genes in CPP are different from those differentially methylated in normal puberty. It might be that in CPP there are different genomic regions that become differentially methylated and that these regions remain epigenetically silent in normal puberty. In fact, only one DMR containing the promoter of *CUTA* was found to be more methylated in CPP patients than in pre-pubertal controls. This gene, mapped to chromosome 6p21.32, encodes a protein of 136 amino acids that plays a role in anchoring of acetylcholinesterase to neuronal membranes in the human brain [44]. The protein CUTA seems to be also involved in promoting proliferation and survival of glial cells [45]. The *CUTA* gene has not been implicated before in the regulation of pubertal development.

Changes in methylation of the two precocious puberty imprinted genes, *MKRN3* and *DLK1*, could represent an interesting causal mechanism of sporadic and familial CPP. However, our results showing a normal methylation status of both genes exclude this potential mechanism as an underlying cause of CPP in our patients. It remains possible that the study of a much larger population of girls with CPP may provide evidence for such a relationship, or that the identification of novel mutations able to alter gene methylation patterns proved to be a causative factor [46–49].

## Conclusion

By demonstrating a widespread pattern of DNA hypermethylation associated with normal and precocious puberty in girls, our results suggest that an epigenetic mechanism involving a chemical change in DNA architecture contributes to regulating pubertal timing in humans. Because these hypermethylation patterns involve several genes, the compelling possibility emerges that the net outcome of these alterations is a modified output from networks controlling the pubertal process. The overrepresentation of *ZNFs* among genes affected by differential methylation and the recent demonstration of an involvement of *ZNFs* in the central control of female puberty in monkeys suggest that *ZNFs* may provide a major regulatory pathway linking DNA methylation to the control of human puberty.

## Methods

### Hormone assays

Serum LH, FSH, and E2 levels were measured by ultrasensitive methods, immunofluorometric assay—IFMA (AutoDELFIA, Turku, Finland), or electrochemiluminometric assay—ECLIA (Cobas e601, Roche Diagnostics, USA), with good correlation among them. The inter-assay and intra-assay coefficients of variation were 5% or less. The hormonal profile was assessed by IFMA (patients) or ECLIA (control group). For the acute GnRH stimulation test, serum LH was measured at − 15, 0, 15, 30, 45, and 60 min after i.v. administration of 100 μg of GnRH. Basal LH levels > 0.6 IU/L (IFMA) or 0.2 IU/L (ECLIA) were considered as pubertal levels, and a GnRH-stimulated LH peak > 6.9 IU/L (IFMA) or 5.0 IU/L (ECLIA) were considered as a pubertal response [50, 51]. The LH level measured 2 h after the first administration of leuprorrelin depot 3.75 mg > 10 IU/L (IFMA) or 5.0 IU/L (ECLIA) was considered pubertal [51, 52].

### Sample preparation and quality control

Genomic DNA was extracted from peripheral blood leukocytes using standard procedures. DNA quality and quantity were assessed by NanoDrop (Thermo Fisher Scientific), Qubit (Thermo Fisher Scientific), and electrophoresis on 1% agarose gel. The bisulfite-converted DNA (EZ DNA Methylation kit, Zymo Research) was hybridized in the Human Methylation 450 BeadChip microarray (HM450K, Illumina), following the Illumina Infinium HD methylation protocol. We used RnBeads tools to evaluate the quality of our data, and all samples provided high-quality data [53]. Briefly, experimental quality control was performed using the microarray positive and negative control probes for staining, hybridization, extension, target removal, bisulfite conversion, specificity, and non-polymorphic sites.

Data were extracted by the iScan SQ scanner (Illumina) using GenomeStudio software (v.2011.1), with the methylation module v.1.9.0, into IDAT files.

Probes were annotated using GRCh37/hg19 coordinates from UCSC regarding genomic positions and features (FDb.InfiniumMethylation.hg19 package), with additional annotations to identify probes that exhibit multiple alignments in the genome for posterior exclusion.

Methylation levels of the CpG sites were calculated as beta values, which range continuously from 0 (unmethylated) to 1 (fully methylated) (http://www.illumina.com).

### Differential methylation analyses

These analyses were performed in the R environment using Bioconductor packages (http://www.bioconductor.org).

The RnBeads package was applied to the dataset [53]. Non-specific probes (*n* = 28,076) were removed due to the high likelihood of cross-hybridization. Background was corrected using the Noob method, which is based on a normal-exponential convolution using out-of-band probes [54]. Normalization of signal intensities values from probes types I and II was performed using SWAN method (Additional file 9), which adjusts the intensities based on a quantile approach [55].

Technical effects and cell blood composition were corrected using default parameters from RnBeads [56]. An expected association between surrogate variables and the age at the time of blood collection was identified, but correction was not applied because these variables are related to the study design (Additional file 10). The clinical treatment for CPP with GnRH analogue did not act as a co-variable.

After the pre-processing step, 443,042 CpG sites were analyzed in pairwise comparisons (pre-pubertal versus pubertal controls, familial CPP cases versus pre-pubertal controls, and familial CPP cases versus pubertal controls).

To identify DMSs, hierarchical linear models from the limma software package followed by a fitting based on the Bayes statistics was applied to *M* values (log of beta values) [57]. CpG sites presenting a FDR < 0.05 and methylation differences greater than 10% were considered as the most significant and selected for further analysis.

DMRcate was applied to identify DMRs, defined as a 300 nucleotides sequence with at least seven CpG sites presenting methylation changes in the same direction [58]. Genomic regions with FDR < 0.05 and mean methylation differences greater than 5% were considered the top ones.

### In silico analyses

Functional enrichment analyses were performed on the Web-based Gene Set Analysis Toolket (WebGestalt) using the whole genome as background [59]. Features with adjusted *p* value < 0.05 provided by the Benjamini–Hochberg multiple test were considered significant. We also used the GSEA program to search for statistically significant associations between a defined set of genes and biological states [60].

### Methylation analyses of *MKRN3* and *DLK1 loci*

Bisulfite-converted DNA samples of all patients and controls were studied using the TaqMan Allele-Specific Methylated Multiplex Real-Time Quantitative Polymerase Chain Reaction to analyze the methylation status at *MKRN3*:TSS-DMR and *MEG3/DLK1*:IG-DMR [61].

### Nonhuman primates

The MBH of female rhesus monkeys (*Macaca mulatta*) was obtained through the Oregon National Primate Research Center (ONPRC) Tissue Distribution Program. The animals were classified into different stages of pubertal development according to the criteria proposed by Watanabe and Terasawa [62]. Early juvenile (EJ) animals were 9 months to 1.8 years of age, late juvenile (LJ) were 2–2.9 years of age, and pubertal (Pub) were 3.1–4 years old. Plasma LH levels at these ages, measured using a different set of animals (*n* = 10/group), were 2.59 ± 0.97 (EJ), 3.88 ± 0.92 (LJ), and 6.48 ± 1.64 (Pub) ng/ml, respectively. The MBH was dissected by making a rostral cut along the posterior border of the optic chiasm, a caudal cut immediately in front of the mammillary bodies, and two lateral cuts half-way between the medial eminence and the hypothalamic sulci, as previously reported [63]. The tissue fragments were frozen in liquid nitrogen and stored at − 80 °C until RNA extraction.

### RNA extraction and quantitative (q) PCR

Total RNA was extracted from the MBH of female rhesus monkeys using the RNeasy mini kit (Qiagen, Valencia, CA). DNA contamination was removed by on-column digestion with DNAse using the Qiagen RNase-free DNase kit (Qiagen, Valencia, CA). RNA concentrations were determined by spectrophotometric trace (Nanodrop, ThermoScientific, Wilmington, DE). Total RNA (500 ng) was transcribed into cDNA in a volume of 20 μl using 4 U of Omniscript reverse transcriptase (Qiagen, Valencia, CA). mRNA was measured using the SYBR GreenER™ qPCR SuperMix system (Invitrogen, Carlsbad, CA). Amplification primers were designed using the PrimerSelect tool of DNASTAR 14 software (Madison, WI) on the NCBI online Primer-Blast program (Additional file 11). PCR reactions were performed in a volume of 10 μl (1 μl of diluted cDNA, 5 μl of SYBR GreenER™ qPCR SuperMix, and 4 μl of primers mix; 1 μM of each gene-specific primer). The PCR conditions used were 5 min at 95 °C, 40 cycles of 15 s at 95 °C, and 60 s at 60 °C. Formation of a single SYBR Green-labeled PCR amplicon was confirmed by subjecting each PCR reaction to a three-step melting curve analysis (15 s at 95 °C, 1 min at 60 °C, ramping up to 95 °C at 0.5 °C/s, detecting every 0.5 s, and ending with 15 s at 95 °C). The qPCR reactions were performed using a QuantStudio 12 K Real-Time PCR system (Thermo Fisher, Waltham, MA), and a QuantStudio 12 K Flex software (Thermo Fisher, Waltham, MA) was used to detect threshold cycles (CTs). Standard curves were constructed by serially diluting (1/2 to 1/512) a pool of cDNAs derived from a mix of equal amounts of cDNA from each sample. The mRNA content of each sample was estimated by referring the corresponding CTs to the relative standard curve, and the values obtained were normalized for procedural losses using glyceraldehyde-3-phosphate dehydrogenase (GAPDH) mRNA as the normalizing unit.

## Additional files

Additional file 1: Gene ontology of the genes related to the top DMRs between pre- and post-pubertal healthy girls. (XLSX 12 kb)

Additional file 2: Most significant transcription factors targeting differentially methylated genes between pre- and post-pubertal healthy girls identified by GSEA. (DOCX 12 kb)

Additional file 3: Volcano plot of differences in DNA methylation between CPP cases (*n* = 10) and pre-pubertal healthy girls (*n* = 15). Each point represents a CpG site (*n* = 443,042) with mean methylation difference on the x-axis and − log10 of FDR on the y-axis. Negative methylation differences indicate hypomethylation and positive differences hypermethylation in the CPP cases compared to the pre-pubertal controls. Green dots represent significantly differentially methylated CpGs (*n* = 417, FDR < 0.05, mean DNA methylation difference > 10%). (TIFF 168 kb)

Additional file 4: Differentially methylated CpG sites between CPP and pre-pubertal control groups, with methylation differences > 10% and FDR < 0.05. (XLSX 50 kb)

Additional file 5: Volcano plot of differences in DNA methylation between CPP cases (*n* = 10) and pubertal healthy girls (*n* = 18). Each point represents a CpG site (*n* = 443,042) with mean methylation difference on the x-axis and − log10 of FDR on the y-axis. Negative methylation differences indicate hypomethylation and positive differences hypermethylation in the CPP cases compared to the pubertal controls. Green dots represent significantly differentially methylated CpGs (*n* = 605, FDR < 0.05, mean DNA methylation difference > 10%). (TIFF 175 kb)

Additional file 6: Differentially methylated CpG sites between CPP and pubertal control groups, with methylation differences > 10% and FDR < 0.05. (XLSX 71 kb)

Additional file 7: Methylation index of the DLK1 and MKRN3 loci in healthy and CPP girls determined by Allele-Specific Methylated Multiplex Real-Time Quantitative Polymerase Chain Reaction. (DOCX 12 kb)

Additional file 8: a) Expression of *ZNFs* selected from a group of *ZNFs* hypermethylated in CPP assessed in the MBH of female rhesus monkeys undergoing puberty. b) Lack of significant changes in *MKRN3* and *DLK1* expression between the early juvenile and peripubertal phases of monkey puberty. The mRNA levels obtained were expressed as fold-change with regard to the values observed in the EJ group. Bars represent mean ± s.e.m. (*n* = 4–7/group) (* = *p* < 0.05; vs EJ group; one-way ANOVA-SNK test). EJ, early juvenile; LJ, late juvenile; PUB, peripubertal. (TIFF 934 kb)

Additional file 9: Normalization of signal intensities values from the Infinium I and II probes by the SWAN method, with beta value on the x-axis and density on the y-axis. (TIFF 151 kb)

Additional file 10: Heatmap displaying the results of permutation tests performed for associations of the co-variables, as given by the RnBeads package. Pink boxes represent significant *p*-values (*p* < 0.01) and blue boxes represent non-significant *p*-values. (TIFF 265 kb)

Additional file 11: Primers used to measure mRNA levels by qPCR in the hypothalamus of female rhesus monkeys. (DOCX 13 kb)

## Abbreviations

B: Breast Tanner stage
BMI: Body mass index
CpG: Cytosine–phosphate–guanine
CPP: Central precocious puberty
DMR: Differentially methylated region
DMS: Differentially methylated site
DNAm: DNA methylation
E2: Estradiol
ECLIA: Electrochemiluminometric assay
EJ: Early juvenile
ER: Estrogen receptor
FDR: False discovery rate
GnRH: Gonadotropin-releasing hormone
GSEA: Gene Set Enrichment Analysis
IFMA: Immunofluorometric assay
IG: Intergenic
LJ: Late juvenile
MBH: Medial basal hypothalamus
mRNA: Messenger RNA
PAR: Pseudoautosomal region
Pub: Pubertal
TSS: Transcription start site
ZNF: Zinc finger
