# Supplementary material for: Methylome profiling of healthy and central precocious puberty girls
Source: Clin Epigenetics. 2018 Nov 22;10:146. doi: 10.1186/s13148-018-0581-1 (PMC6251202; doi:10.1186/s13148-018-0581-1)
Supplement: Supplementary file 2 — Most significant transcription factors targeting differentially methylated genes between pre- and post-pubertal healthy girls identified by GSEA. (DOCX 12 kb) [file 13148_2018_581_MOESM2_ESM.docx]

| **Transcription factor Gene symbol** | **Genes with the transcription factor binding site** | ***p*-value** |
| --- | --- | --- |
| GGGCGGR_*SP1* | *SMARCA1, POU5F1, PTCHD1, GPC3, PRDM8, SUV39H1, CASK, FLNA, OTUD5, TFE3, TAF1, CPT1B, HTATSF1, SLC25A5, RPS6KA3, C1GALT1C1, LONRF3, NDUFB11, RBM10, NHS, XIAP, PGRMC1, UBE2A, SYN1, ALOX12, ATP6AP1, GPRASP2, LAGE3* | 2.15E-9 |
| GGGAGGRR_*MAZ* | *SMARCA1, POU5F1, PTCHD1, GPC3, PRDM8, SUV39H1, CASK, TEF3, TAF1, CPT1B, FAM155B, OPHN1, ELF4, TSPAN7, FGF13, DUSP9, GNAS, CNKSR2, MSL3, NLGN2, SASH3* | 5.04E-7 |
| TGACCTY_*ERR1* | *SMARCA1, POU5F1, HTATSF1, SLC25A5, FAM155B, OPHN1, ELF4, TSPAN7, TAZ, HMGB3, HS6ST2, PGK1, PRDM9, NR0B1* | 7.04E-7 |
| CAGGTG_*E12* | *PTCHD1, HTATSF1, SLC25A5, RPS6KA3, C1GALT1C1, LONRF3, FLNA, FAM155B, OPHN1, FGF13, DUSP9, GNAS, CNKSR2, TAZ, LY6G5C, GABRQ, ZIC3, KLHL34, RBM3, SMS, DNASE1L1* | 2.08E-6 |
| GATTGGY_*NFY* | *GPC3, PRDM8, SUV39H1, OTUD5, NDUFB11, RBM10, GABRQ, ZIC3, MSL3, PIGA, ZNF597, FGFR2, CETN2, NSDHL* | 2.43E-6 |
| *ER* | *SMARCA1, POU5F1, HTATSF1, ELF4, HMGB3, KLHL34, FGFR2* | 5.09E-6 |
| CTGCAGY_UNKNOWN | *PTCHD1, GPC3,CASK, RPS6KA3, ELF4, TSPAN7, FGF13, DUSP9, NLGN2, SASH3, GABRQ* | 6.16E-6 |
| TGGAAA_*NFAT* | *PTCHD1,CASK, TEF3, RPS6KA3, C1GALT1C1, OTUD5, ELF4, FAM155B, OPHN1, FGF13, GNAS, TAZ, HS6ST2, GABRQ, FAM120C, GRB10, PDK3* | 1.02E-5 |
| GCCATNTTG_*YY1* | *RPS6KA3, NDUFB11, RBM10, RBM3, FAM120C, IRAK1, ZNF41, RPL10* | 1.87E-5 |
| *ZF5* | *HTATSF1, NDUFB11, RBM10, NHS, TSPAN7, FGF13* | 4.34E-5 |

**Additional file 2.** Most significant transcription factors targeting differentially methylated genes between pre- and post-pubertal healthy girls identified by GSEA
