# Supplementary figures and images for: Methylome profiling of healthy and central precocious puberty girls
Source: Clin Epigenetics. 2018 Nov 22;10:146. doi: 10.1186/s13148-018-0581-1 (PMC6251202; doi:10.1186/s13148-018-0581-1)

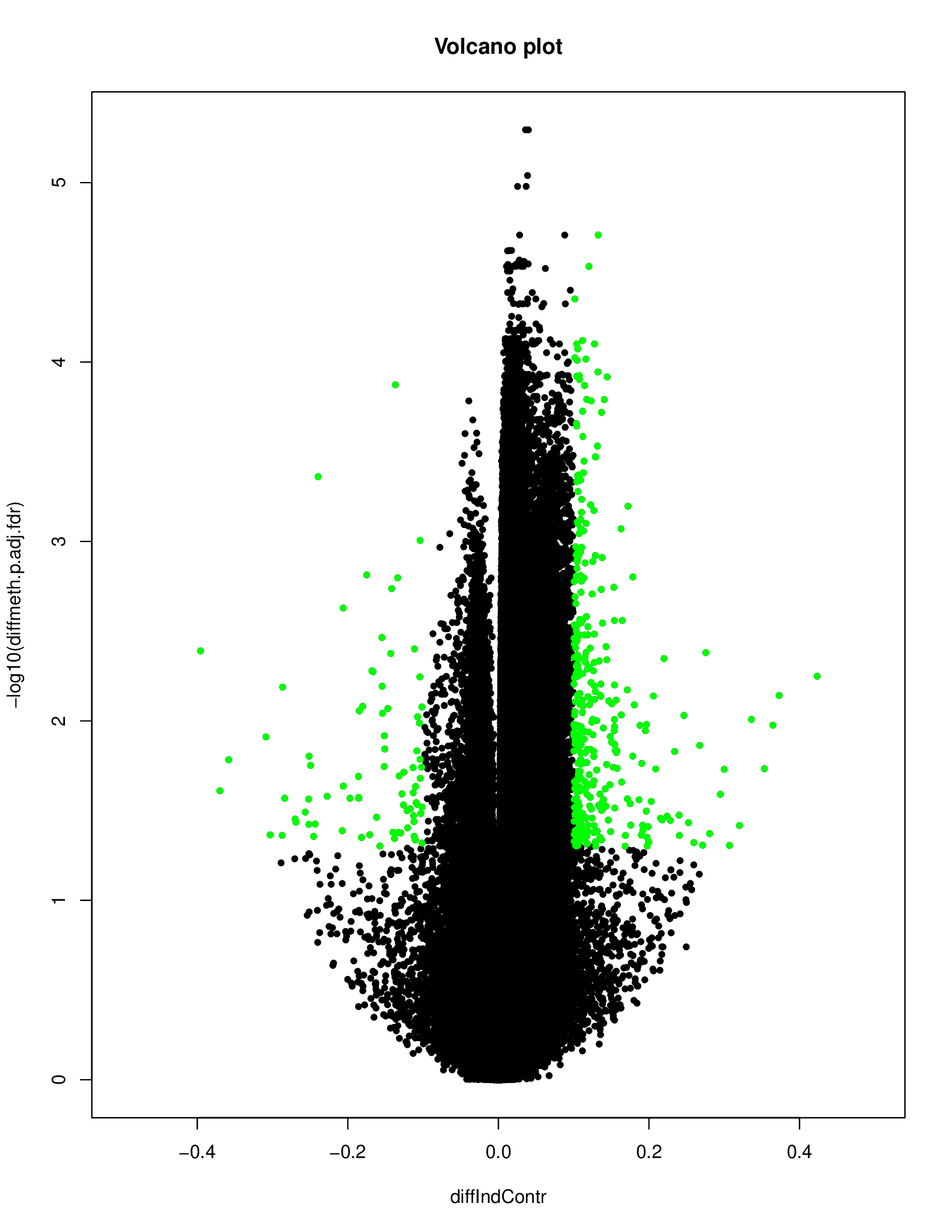

Supplement: Supplementary file 3 — Volcano plot of differences in DNA methylation between CPP cases (n = 10) and pre-pubertal healthy girls (n = 15). Each point represents a CpG site (n = 443,042) with mean methylation difference on the x-axis and − log10 of FDR on the y-axis. Negative methylation differences indicate hypomethylation and positive differences hypermethylation in the CPP cases compared to the pre-pubertal controls. Green dots represent significantly differentially methylated CpGs (n = 417, FDR < 0.05, mean DNA methylation difference > 10%). (TIFF 168 kb) [file 13148_2018_581_MOESM3_ESM.tiff]

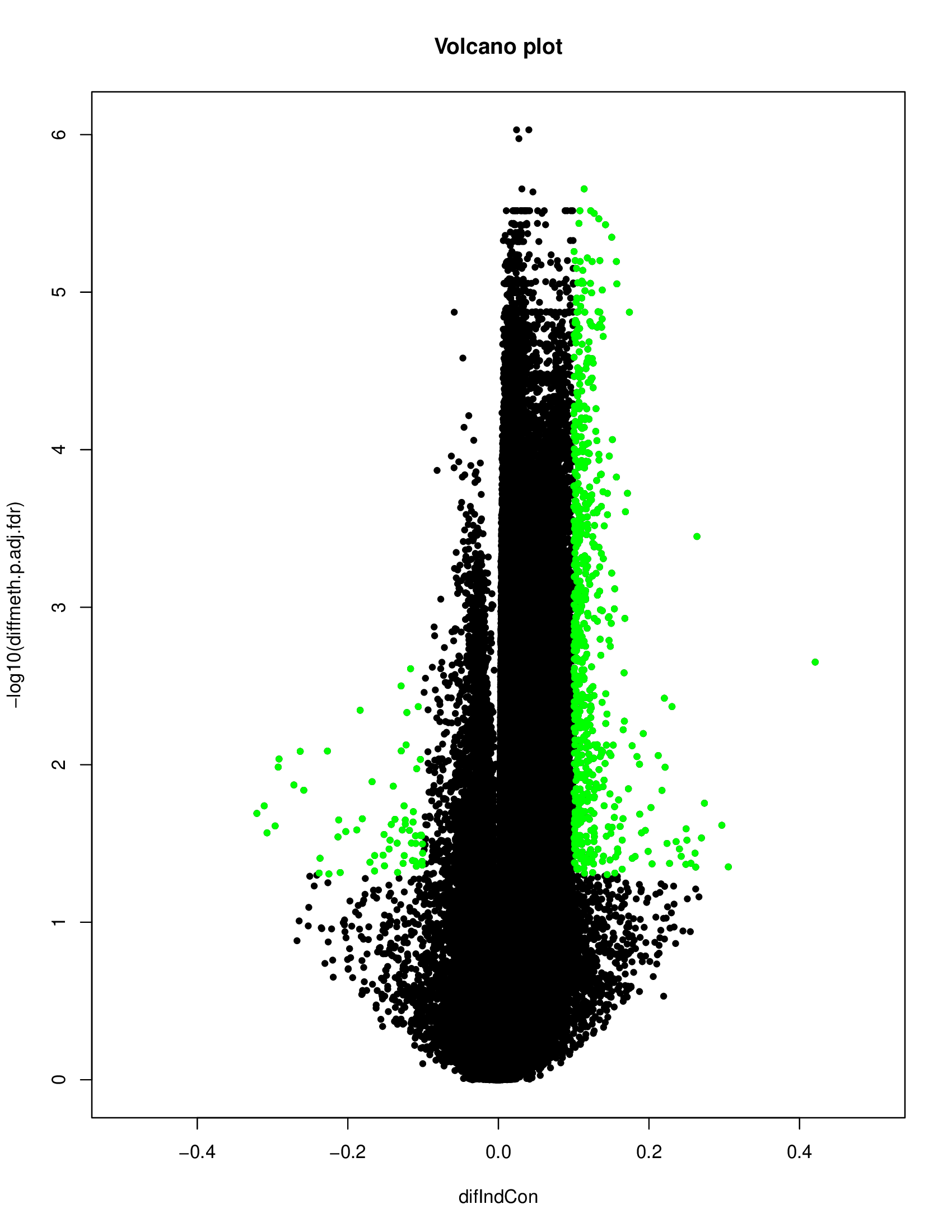

Supplement: Supplementary file 5 — Volcano plot of differences in DNA methylation between CPP cases (n = 10) and pubertal healthy girls (n = 18). Each point represents a CpG site (n = 443,042) with mean methylation difference on the x-axis and − log10 of FDR on the y-axis. Negative methylation differences indicate hypomethylation and positive differences hypermethylation in the CPP cases compared to the pubertal controls. Green dots represent significantly differentially methylated CpGs (n = 605, FDR < 0.05, mean DNA methylation difference > 10%). (TIFF 175 kb) [file 13148_2018_581_MOESM5_ESM.tiff]

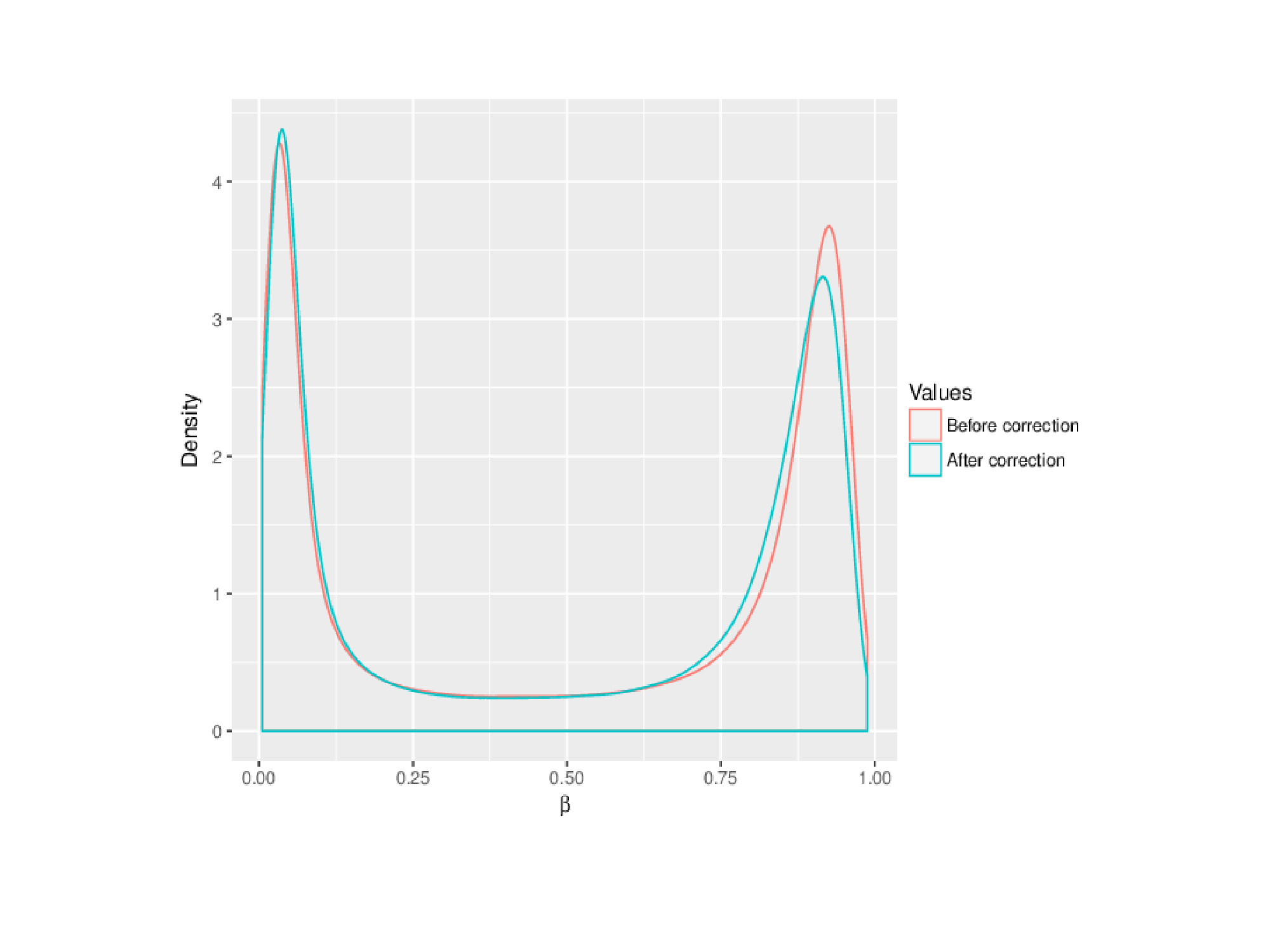

Supplement: Supplementary file 9 — Normalization of signal intensities values from the Infinium I and II probes by the SWAN method, with beta value on the x-axis and density on the y-axis. (TIFF 151 kb) [file 13148_2018_581_MOESM9_ESM.tiff]

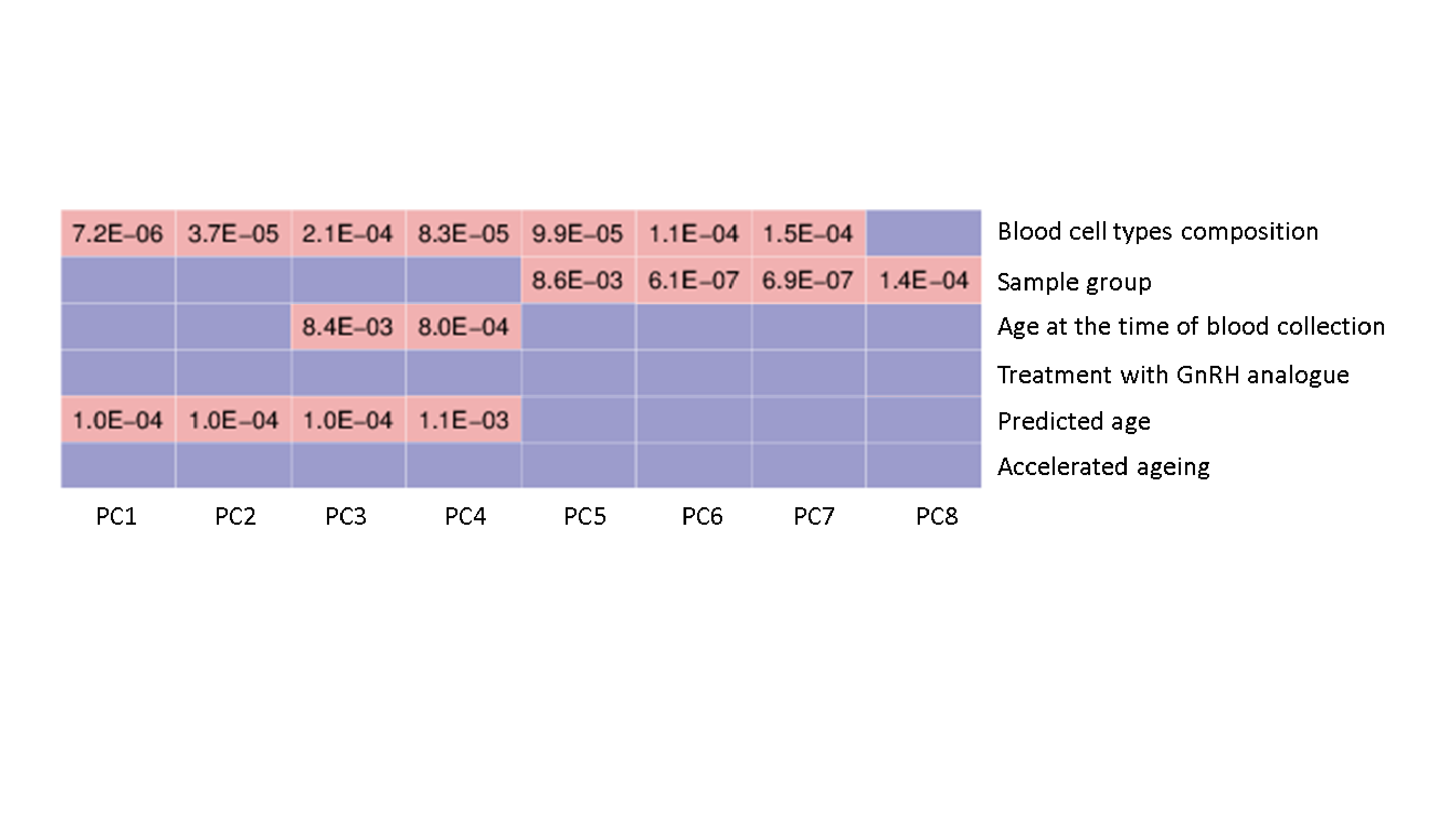

Supplement: Supplementary file 10 — Heatmap displaying the results of permutation tests performed for associations of the co-variables, as given by the RnBeads package. Pink boxes represent significant p-values (p < 0.01) and blue boxes represent non-significant p-values. (TIFF 265 kb) [file 13148_2018_581_MOESM10_ESM.tiff]
