# Supplementary material for: Methylome profiling of healthy and central precocious puberty girls
Source: Clin Epigenetics. 2018 Nov 22;10:146. doi: 10.1186/s13148-018-0581-1 (PMC6251202; doi:10.1186/s13148-018-0581-1)
Supplement: Supplementary file 7 — Methylation index of the DLK1 and MKRN3 loci in healthy and CPP girls determined by Allele-Specific Methylated Multiplex Real-Time Quantitative Polymerase Chain Reaction. (DOCX 12 kb) [file 13148_2018_581_MOESM7_ESM.docx]

**Additional file 7.** Methylation index of the *DLK1* and *MKRN3* *loci* in healthy and CPP girls determined by Allele-Specific Methylated Multiplex Real-Time Quantitative Polymerase Chain Reaction

|  | **Methylation index (%)** | |
| --- | --- | --- |
|  | ***DLK1-MEG3:*IG-DMR** | ***MKRN3:*TSS-DMR** |
| **Pre-pubertal control group** | | |
| Mean (SD) | 52 (±1,7) | 48 (±4,7) |
| Normal range | 49-55 | 39-57 |
| **Pubertal control group** | | |
| Mean (SD) | 51 (±1,4) | 50 (±7,3) |
| Normal range | 48-54 | 35-65 |
| **Familial CPP group** |  |  |
| Index cases |  |  |
| 1 | 54 | 46 |
| 2 | 51 | 51 |
| 3 | 51 | 51 |
| 4 | 52 | 37 |
| 5 | 52 | 38 |
| 6 | 49 | 41 |
| 7 | 51 | 48 |
| 8 | 51 | 56 |
| 9 | 49 | 36 |
| 10 | 49 | 44 |
| IG = intergenic; SD = standard deviation; TSS = transcription start site | | |
