# Supplementary material for: Methylome profiling of healthy and central precocious puberty girls
Source: Clin Epigenetics. 2018 Nov 22;10:146. doi: 10.1186/s13148-018-0581-1 (PMC6251202; doi:10.1186/s13148-018-0581-1)
Supplement: Supplementary file 11 — Primers used to measure mRNA levels by qPCR in the hypothalamus of female rhesus monkeys. (DOCX 13 kb) [file 13148_2018_581_MOESM11_ESM.docx]

**Additional file 11**. Primers used to measure mRNA levels by qPCR in the hypothalamus of female rhesus monkeys

| **GNRH** |  |
| --- | --- |
| mkGNRHF | GGCAGAAACCCAACACTTCG |
| mkGNRHR | CACTCCTTCTGGCCCAATAGA |
| **KISS1** |  |
| mkKISS1F | AGAAAAGGTGGCCTCTGTGGA |
| mkKISS1R | AGGCTCTGCTCCCACGG |
| **TAC3** |  |
| mkTAC3F | TGGGGCTGTCTGTAAGGAGC |
| mkTAC3R | CCCTCCAGAGATGAGTGGCT |
| **ZFP57** |  |
| mkZFP57F | GGAGGGAGGCATGGGTGAAG |
| mkZFP57R | AGCTTGGTGATTAGCTCTGGCT |
| **ZNF597** |  |
| ZNF597 F | TGTTTGGAGGATGCGGCCTT |
| ZNF597 R | CTGGGTAAGGGACAAGGGGTG |
| **ZNF41** |  |
| ZNF41 F | GAGGGACGTGGCAGCTCAT |
| ZNF41 R | CACTGAGAGCAGGTGGCTGT |
| **ZNF707** |  |
| ZNF707 F | TCAGGACCCGGTTGTGTATCC |
| ZNF707 R | CTGGGGAAACACAGCGCCAA |
| **ZNF331** |  |
| ZNF331 F | AGGTCAGCTCTAGCCTCTCA |
| ZNF 331 R | TACGTCGGCGAATGTCACCA |
| **PRDM8** |  |
| PRDM8 F | CGGGTTCCAGGTGGCCTTAT |
| PRDM8 R | GGGATGTCGCAGGTGGTGTA |
| **ZNF251** |  |
| mkZNF251F | TGGGAGCTGAGGAACCAGAG |
| mkZNF251R | ACCCTCATGGCACCATGCTT |
| **RNF113A** |  |
| mkRNF113AF | AGAAGCGGGCGACTCACAAT |
| mkRNF113AR | ACAGCTGTCGCCCCCATATC |
| **ZDBF2** |  |
| mkZDBF2F | TGCAGCACCACCATATCACT |
| mkZDBF2R | CCACTTCAGATGATGACCCAGT |
| **ZDHHC9** |  |
| mkZDHHC9F | CTCGGGCGCTACCAGATGAA |
| mkZDHHC9R | GCGCTCCACACAGTTGTCAC |
| **MKRN3** |  |
| mkMKRN3F | CAAGAGTAAATGTGAGTGAAAGGAGA |
| mkMKRN3R | TGAAGGGGAAGGAGGGAAAGAACAA |
| **DLK1** |  |
| mkDLK1F | TGTGTGACCAGTGCGTGACC |
| mkDLK1R | CAGGGGCACAGGAGCATTCA |
| **GAPDH** |  |
| mkGAPDHF | AAGGGCATCCTGGGCTACA |
| mkGAPDHR | GAAGAGTGGGTGTCGCTGTTG |
